# Responses of the summer Asian-Pacific zonal thermal contrast and the associated evolution of atmospheric circulation to transient orbital changes during the Holocene

**DOI:** 10.1038/srep35816

**Published:** 2016-10-25

**Authors:** Dong Xiao, Ping Zhao, Yue Wang, Xiuji Zhou

**Affiliations:** 1 State Key Laboratory of Severe Weather, Chinese Academy of Meteorological Sciences, Beijing 100081, China; 2 Institute of Climate Systems, Chinese Academy of Meteorological Sciences, Beijing 100081, China; 3 Collaborative Innovation Center on Forecast and Evaluation of Meteorological Disasters, Nanjing University of Information Science and Technology, Nanjing, 210044, China; 4 State Key Laboratory of Marine Geology, Tongji University, Shanghai, 200092, China

## Abstract

This study investigates the response of large-scale atmospheric circulation over the Asian-Pacific sector and precipitation over eastern China to transient orbital changes during the Holocene summer using an intermediate-complexity climate model. Corresponding to variations in the incoming solar radiation, the eddy sea level pressure (SLP) exhibited an out-of-phase relationship between the North Pacific and the Eurasian landmass that was similar to the present-day Asia-Pacific Oscillation (APO) pattern and was defined as the paleo-APO. Its index presented an increasing trend, which implies the enhancement of a zonal thermal contrast between Asia and the North Pacific. Associated with the strengthening of the paleo-APO was the westward shift in North Pacific high pressure. Accordingly, there was less/more summer precipitation over both the middle reach of the Yangtze River and Southwest China/over North China. The high-resolution stalagmite δ^18^O records further support this decrease in the model precipitation. Along with the strengthening of paleo-APO from the early Holocene to the present, the eddy SLP anomalies exhibited a decreasing/increasing trend over the Eurasian landmass/the North Pacific, with a phase change of approximately 4.5 ka BP, and they both moved westward. Meanwhile, a less rainfall belt over eastern China exhibited northward propagation from southern China.

The East Asian summer monsoon (EASM) is an important component of global climate systems and plays significant roles in global energy and hydrological cycles. Therefore, it is viewed as the life blood providing the key precipitation for agriculture^1^. However, some extreme variations in the EASM also cause harmful drought and flooding events^2^. In particular, under recent global warming, the EASM experienced a weakening trend, with severe droughts over northern China and disastrous floods over the Yangtze River valley^3^. Therefore, understanding the natural variability of EASM and predicting its long-term changes in a warming climate are helpful for disaster mitigation.

The Holocene is another significant warming period that occurred since the last glacial period. Studies of EASM during the Holocene may help us understand the mechanisms responsible for the natural variability of EASM under a warming scenario, which has been of increasing interest during the past decade^4^,^5^,^6^,^7^,^8^,^9^,^10^,^11^,^12^. Some studies of high-resolution and precisely dated contemporaneous speleothem δ^18^O records from caves have indicated a long-term downward trend of the EASM precipitation during the Holocene^13^,^14^. This trend may be a response to the decreasing solar insolation over the Northern Hemisphere (NH). However, the associated mechanisms remain unknown.

In the summer, a remarkable tropospheric zonal thermal contrast arises between the warmer Eurasian continent and the cooler North Pacific or Indian Ocean. Some in-depth studies have explored the variations in the thermal contrast and their links with EASM and precipitation on multiple time scales^3^,^15^,^16^,^17^,^18^. Recently, a large-scale summertime teleconnection pattern of the tropospheric temperature over the extratropical NH, with opposite anomalous centers between Asia and the North Pacific, was proposed as the Asian-Pacific Oscillation (APO)^19^. A higher (lower) APO index often represents a stronger (weaker) thermal contrast between the Eurasian continent and the North Pacific Ocean. The APO index also sufficiently represents the long-term changes in the EASM rainfall over the past century^20^ and millennium^21^,^22^,^23^,^24^.

Climate models have been widely used to improve our understanding of the mechanisms of Holocene climate changes. In particular, the first, second and third phases of the Paleoclimate Modeling Inter-comparison Project (PMIP1, PIMP2, and PIMP3) focused on the Mid-Holocene, 6 ka before the present (ka BP). Some equilibrium simulations (viz., “snapshot”) were applied to compare the characteristics and mechanisms of climate change over China between the Mid-Holocene and the present, in which the concentration of atmospheric CO2 was held at 280 ppm^25^,^26^,^27^,^28^,^29^,^30^. Zhou and Zhao^31^ examined the APO variation and associated summer precipitation anomalies using the CCSM3 simulation in PIMP2. Their results presented a pattern of northwest-southeast inclination in the Mid-Holocene, which mainly results from the incoming solar radiation differences caused by changes in the orbital parameters. Moreover, to understand climatic evolutions over the entire Holocene period, some transient simulations under the forcing of orbital changes were also performed with different complexity climate models and accelerated schemes^32^,^33^,^34^,^35^,^36^,^37^,^38^,^39^,^40^.

Although climate simulations for the Holocene have made great progress, a number of questions remain unanswered. For example, how do large-scale circulations respond to the NH decreasing incoming solar radiation during the Holocene summer? Does this response cause a significant difference between land and ocean? If so, how do they affect the EASM hydrological climate? What are the evolving characteristics of atmospheric circulation in the Holocene? With these questions in mind, we use an intermediate-complexity climate model to perform a transient simulation with an acceleration factor of 10 years, under the orbital changes during the past 10 ka BP, and investigate an APO-like large-scale teleconnection over the Asian-Pacific sector and the associated rainfall anomalies over eastern China. Meanwhile, some simulated results are compared with the proxy data. The remainder of this paper is organized as follows. The UVic Model, data, and methods are introduced in the next section. In the result section, the responses of large-scale circulations to orbital changes are analyzed and compared with proxies; and the EASM circulation and hydrological climate evolutions associated with the APO-like large-scale teleconnection during the Holocene are investigated. A summary and a further discussion are provided in the last section.

## Model, Experiment, Forcing, Data and Methods

### Model and transient experiment

The UVic Model (version 2.9) developed by the University of Victoria, Canada couples the atmospheric, oceanic, sea ice, and land surface model components, and it has a resolution of 3.6° × 1.8° in longitude and latitude. The atmospheric model comprises a single layer (that is, a vertically integrated energy-moisture balance model). The ocean component comes from version 2.2 of the GFDL Modular Ocean Model, with 19-vertical levels. The land surface model employs the MOSES (Met Office Surface Exchange Scheme) model. The UVic Model is often used as a tool for understanding the physical process and feedback in the climate system on long timescales, and is widely used in paleoclimate, present climate, and future climate studies^41^,^42^,^43^,^44^,^45^. This model could reasonably reproduce temperature, precipitation, and ocean circulation, with relatively small errors, even compared with the coupled General Circulation Model (GCM)^42^. However, the internal high-frequency variations of almost all of the variables on interannual and interdecadal timescales in the UVic Model are weaker compared with those of the complex atmospheric models^46^. Therefore, the UVic Model is mainly used to investigate the climate processes and feedback on long timescales^42^. In this study, we also perform five experiments with different initial fields to test the uncertainty of the model results. An ensemble mean of variables is used, and the spreads of the time series from the ensemble mean are identified.

Because of limited computer capabilities, simulations with acceleration factors between 10 and 100 have been applied to long-term climate studies, and they yield similar results^47^. In the present study, following Kutzbach *et al*.^35^, we begin with orbital parameters at 10 ka BP, and they are advanced by 10 years at the end of each model year (that is, with an acceleration factor of 10). The UVic model is spun up for two thousand years before the acceleration and is then integrated for 1001 model years. Sea level air temperature (SLAT), sea level pressure (SLP), and precipitation are used to analyze the climatic variations.

According to the UVic Model, we calculate SLP from the output of SLAT (*T*_*s*_) as follows^42^:


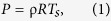


where *R* is the gas constant of dry air (287 J kg^−1^ K^−1^); ρ is the air density; and ρ = *a* + *bT*_*s*_. This relationship between ρ and *T*_*s*_ has been demonstrated as reasonable by the atmospheric reanalysis data of both the National Centers for Environmental Prediction-National Centers for Atmospheric Research (NCEP-NCAR) and the European Centre for Medium-range Weather Forecasts (ECMWF)^42^. In this study, we calculate *a* and *b* using the air temperature and SLP of the 1981–2010 NCEP-NCAR reanalysis.

### Transient Orbital Forcing

**Figure 1:**
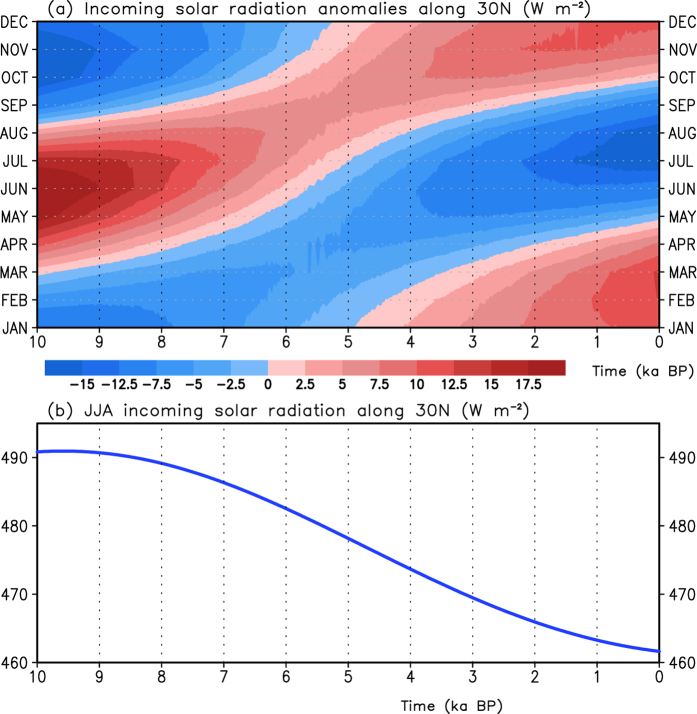
(**a**) Annual cycles of the incoming solar radiation anomaly along 30°N relative to the climatological mean over the past 10 ka and (**b**) the summer incoming solar radiation along 30°N. The abscissa is time before the present (1950 AD), and the ordinate in (**a**) is the month of the year.

Due to the precession effect, the incoming solar radiation is redistributed over months of a year during the past 10 ka. Figure 1a shows the month-year cross section of the transient incoming solar radiation along 30°N that is used in this study^48^,^49^. The incoming solar radiation exhibits a decreasing trend in the warm season (May-August), with a maximum variation exceeding 32.5 W·m^−2^, an increasing trend in the cold season (October-March), with a maximum variation exceeding 27.5 W·m^−2^, and an increase (decrease) before 5 ka and a subsequent decrease (increase) in September (April). The summer incoming solar radiation from June to August along the 30°N (Fig. 1b) presents an obvious declining trend, ranging from 491 to 462 W·m^−2^ during the Holocene. A previous study indicated that the application of different calendars in the paleoclimate simulation could induce artificial phase shifts in solar insolation forcing and climatic responses^50^. Such a “calendar effect” in each month due to the precession might be modified by the fixed angular calendar according to Chen *et al*.^51^. In this study, we focus on the variation in summer (June, July and August, JJA) atmospheric circulation after the above modification.

### Proxy and Reanalysis Data

**Figure 2:**
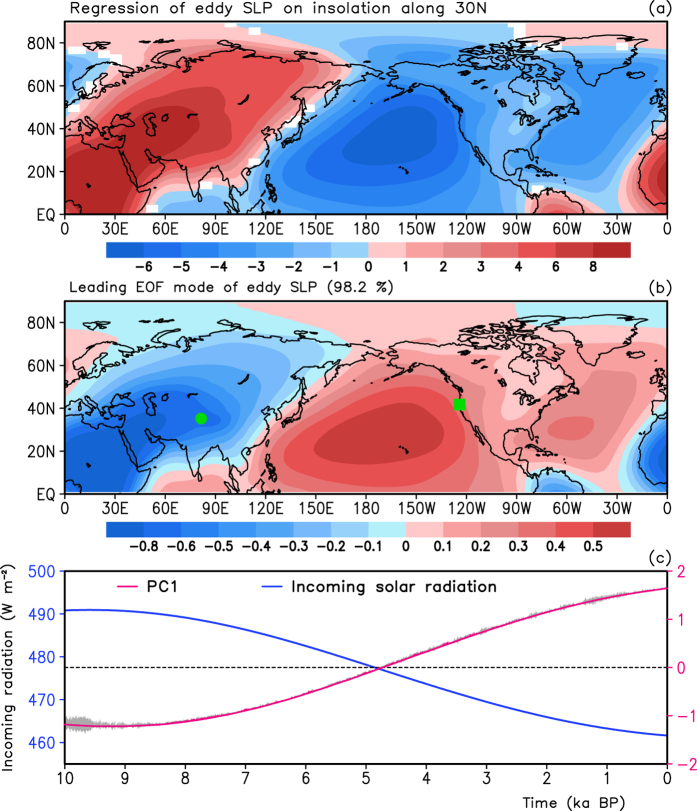
(**a**) Regressed eddy SLP (Unit: Pa/W·m^−2^) on the JJA incoming solar radiation along 30°N, in which values exceeding the 95% confidence level are plotted. (**b**) Leading EOF mode of the eddy SLP, in which the green circle and square indicate the locations of the Guliya ice core and the ODP 1019, respectively. (**c**) The PC1 and JJA incoming solar radiation along 30°N during the past 10 ka BP, in which the gray shaded area represents the spread of the ensemble mean PC1. These figures were generated by the Grid Analysis and Display System (GrADS) Version 2.0.1.oga.1 Copyright (**c**) 1988–2011 by Brian Doty and the Institute for Global Environment and Society (IGES) (ftp://cola.gmu.edu/grads/2.0/old/).

To evaluate the simulated SLAT/sea surface temperature (SST) over the Eurasian continent/the North Pacific (a key region of SLAT/SST anomalies associated with orbital forcing), we employ the oxygen isotope record from the Guliya ice cap (at 81.5°E, 35.2°N) of western China^52^,^53^,^54^ and the reconstructed northern California SST (at 124.9°W, 41.7°N) over the North Pacific^55^,^56^, which have the ability to indicate summer conditions. Their locations are shown in Fig. 2b. The Guliya temperature proxy over the past 130 ka, drilled from the Guliya ice cap, is recorded as a fluctuation in the concentration of oxygen 18 (δ^18^O) (hereinafter Guliya δ^18^O record) in a unit of Vienna Pee Dee Belemnite (VPDB). The monthly concentration of δ^18^O in Guliya is shown to be highly related to the local monthly surface air temperature^57^. Further studies disclosed that the Guliya δ^18^O record could indicate summer temperatures over the Tibetan Plateau during the past 130 ka^44^,^58^. The Northern California SST was reconstructed from the alkenone in the Ocean Drilling Program (ODP) Site 1019 (ODP 1019), with a water depth of 980 m^55^. Generally speaking, the alkenone SST of ODP 1019 represents the annual mean SST. Because the annual mean SST over the region 110°W–120°W, 35°N–45°N in the present-day climate is highly related to the local June–July SST, with a correlation coefficient of 0.82 during 1981–2010 (exceeding the 99.9% confidence level), the annual mean SST over the eastern North Pacific might be used to indicate the equivalent variation in the local June-July averaged SST. Moreover, the annual mean SST is correlated with the summer North Pacific mean SLP over the region 120°W–180°, 15–60°N, with a correlation coefficient of −0.39 (exceeding the 95% confidence level). This correlation is higher than that between the annual mean SST and the winter North Pacific SLP (−0.25, not significant at the 95% confidence level), which suggests that the annual mean SST over the eastern North Pacific could better indicate the summer North Pacific SLP instead of the winter one. Therefore, we employ the alkenone SST of ODP 1019 to indicate the summer (June–July) SST. The ODP sites 1014 and 1016, adjacent to the ODP 1019, could indicate the variation of SLP over the North Pacific and their reconstructed SSTs show a similar low-frequency SST variation^59^. It is concluded that the ODP 1019 SST may also represent the variability in the North Pacific high pressure like the ODP sites 1014 and 1016 SST. Due to a low resolution of the reconstructed SSTs in the ODP sites 1014 and 1016 during the Holocene, we use the reconstructed northern California SST in the ODP 1019 for evaluating the simulated SST over the North Pacific. Furthermore, we also use the reconstructed July temperatures with high temporal resolutions over the middle-west part of North America^60^ and Western Europe (68°22′N, 18°42′E)^61^ (shown in Fig. S1a,b) to further examine the capability of the model.

To evaluate the variation in the model summer precipitation over eastern China, five high-resolution speleothem δ^18^O records at Dongge Cave (108.08°E, 25.28°N)^61^, Lianhua Cave (109.53°E, 29.48°N)^6^, Sanbao Cave (110.43°E, 31.67°N)^7^, Heshang Cave (110.42°E, 30.45°N)^9^, and Jiuxian Cave (109.1°E, 30.57°N)^5^, released on a website from the World Data Center for Paleoclimatology, are employed to compare with the simulated summer precipitation during the Holocene. These records were derived from karst caves in the middle reach of the Yangtze River, one of the key regions of EASM where the Meiyu rain belt appears. Previous studies have argued that the signal of the oxygen isotope composition (δ^18^O) in speleothem represents precipitation variations^63^,^64^,^65^,^66^. Caley *et al*.^67^ proposed that the speleothem δ^18^O in South Asia represents a change in hydrology. Their results suggested that the calcite δ^18^O signal of South Asian speleothem is mainly, but not entirely, controlled by δ^18^O in precipitation changes. Furthermore, δ^18^O in precipitation over South Asia is correlated to the northern tropical Indian Ocean which is one of the important moisture sources of East Asian monsoon precipitation. Moreover, the East Asian speleothem δ^18^O may relate to the ratio of moisture from the Bay of Bengal (BOB) and the Northwest Pacific^68^. In a modern climate, precipitation over eastern China is highly correlated with moisture over the BOB and the Northwest Pacific; that is, a change of δ^18^O in precipitation is the most important factor affecting the variation of the speleothem δ^18^O records in eastern China. Therefore, we use these records as a hydrologic factor and compare them with the most important hydrological factors such as precipitation. Moreover, we also use monthly mean air temperature and SLP of the NCEP-NCAR reanalysis^69^ with a horizontal resolution of 2.5° and the monthly Hadley SST with 1° in both longitude and latitude^70^.

### Statistical Methods

Regression and correlation analyses are used to investigate the relationship between two variables. An Empirical Orthogonal Function (EOF) analysis with area-weighting is employed to examine the leading mode of the eddy SLP. A composite analysis between the higher and lower indices is used to analyze variations in a variable. The confidence tests in this study are all based on a Student’s *t-*test.

## Results

### Response of eddy SLP over the Asian-Pacific sector to incoming solar insolation

Figure 2a shows the regressed model summer eddy SLP (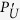) against the incoming solar radiation along 30°N, in which the 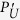 is defined as the difference of SLP from the global zonal mean. The 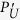 anomaly pattern associated with incoming solar radiation presents a zonal dipole, with positive anomalies over Europe, Asia and North Africa and negative anomalies over the North Pacific Ocean and North America, which indicate a large-scale out-of-phase relationship between Eurasia and the extratropical North Pacific. The maximum positive center is 8 Pa/W·m^−2^, larger compared with the absolute value of the negative center (−5 Pa/W·m^−2^). This result indicates that when the 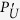 over the East Hemisphere landmass is higher (lower), it is lower (higher) over the North Pacific Ocean and the North America landmass. Furthermore, the decrease in the incoming solar radiation is associated with both a decrease of the 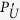 over Eurasia and North Africa and an increase over the North Pacific and North America, which corresponds to a strong thermal contrast between the Eurasian-North African region and the North Pacific-North American region.

In fact, the out-of-phase relationship presented in Fig. 2a is also a major feature of 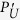 variability. The EOF analysis on the summer eddy SLP shows that the leading EOF mode (Fig. 2b) of the eddy SLP, accounting for 98.2% of the total variance, depicts a zonal out-of-phase structure with negative anomalies over the Eurasian and North African landmass and positive anomalies over the North Pacific, similar to those shown in Fig. 2a. The spatial correlation coefficient between the regressed field (in Fig. 2a) and the leading EOF mode (Fig. 2b) is 0.99 (significant at the 99.9% confidence level). As shown in Fig. 2c, a decreasing trend of the incoming solar radiation is corresponded to an increasing trend in the PC1. This result indicates that the summer 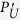 over Eurasia/the North Pacific and North America decreases/increases in the Holocene, which may lead to an anomalous zonal contrast of the 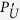 between the Eurasian landmass and the North Pacific Ocean. Moreover, the anomalous pattern of the 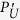 during the Holocene is closely associated with the orbital forcing.

**Figure 3:**
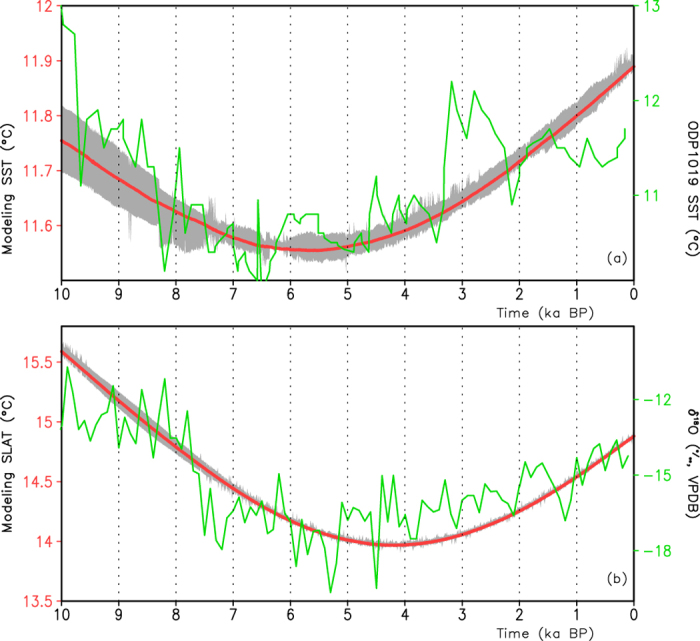
(**a**) Reconstructed SST (unit: °C; green) at the ODP 1019 and simulated June-July SST (unit: °C; red) over the northeastern Pacific (110°W–120°W, 35°N–45°N), in which the gray shaded area represents the spread from the model ensemble mean. (**b**) Same as (**a**) but for the oxygen isotope record in the Guliya ice core (unit: VPDB; green) and the simulated summer SLAT (unit: °C; red) over 80°E–85°E, 33°N–38°N.

To examine the reliability of the simulation, referring to some key regions of the anomalous centers in Fig. 2b, we select reconstructions of the Guliya record and the northern California SST at the ODP 1019. The reconstructed northern California SST displays a low frequent variation, with a decreasing trend from 10 ka to 6.4 ka BP and an increasing trend during the period from 6.4 ka BP to the present (Fig. 3a). A high frequent variation on the century-to-millennium scales is superposed over this low frequent variation. The simulated June–July averaged Northeast Pacific SST also indicates a decrease during the epoch 10–5 ka BP and then an increase from 5 ka BP to the present. The spread of the ensemble mean June–July Northeast Pacific SST varies within small fluctuation, approximately from 0.05 in the early Holocene to 0.02 °C in the middle and late Holocene, which indicates a small uncertainty in the simulation. It is evident that the simulated and reconstructed SSTs over the Northeast Pacific show similar lower frequency variations during the Holocene. Figure 3b displays the curves of the simulated summer Guliya SLAT and the Guliya δ^18^O record during the past 10 ka. The Guliya δ^18^O record, indicating a summer temperature over the Tibetan Plateau, presents a decrease during the period of 10–4.5 ka BP and an increase from 4.5 ka BP to the present. The simulated summer temperature over the Guliya region also shows a similar lower-frequency variation, with a decrease during 10–4.5 ka BP and an increase from 4.5 ka BP to the present. Uncertainties in the ensemble simulations of the Guliya temperature show little spread, which thereby supports a more stable simulation of the UVic Model. Thus, the simulated Guliya SLAT reproduces well the lower-frequency variation of the Guliya δ^18^O record (namely, the summer temperature variation over the Tibet Plateau). Figure S1 shows the simulated and reconstructed July temperatures over the mid-western part of the United States and Western Europe. The simulated July SLAT over the mid-western part of the United States presents a declining trend during the Holocene, which also appears in the reconstructed time series (Fig. S1a). Meanwhile, the July temperature variation over the mid-western part of the United States is similar to that of the North Pacific center of the paleo-APO pattern. Moreover, the reconstructed July temperature over Western Europe also supports the model simulation on the long-time scales (Fig. S1b). The consistency suggests the reliability of the simulated results over Eurasia and the North Pacific.

**Figure 4:**
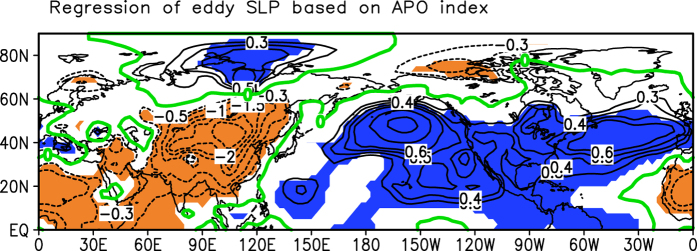
Regression of the eddy SLP (unit: hPa) on the APO index during 1948–2014. The shaded areas are significant at 95% confidence levels and the green contours are zero. This figure is generated by the Grid Analysis and Display System (GrADS) Version 2.0.1.oga.1 Copyright (**c**) 1988–2011 by Brian Doty and the Institute for Global Environment and Society (IGES) (ftp://cola.gmu.edu/grads/2.0/old/).

**Figure 5:**
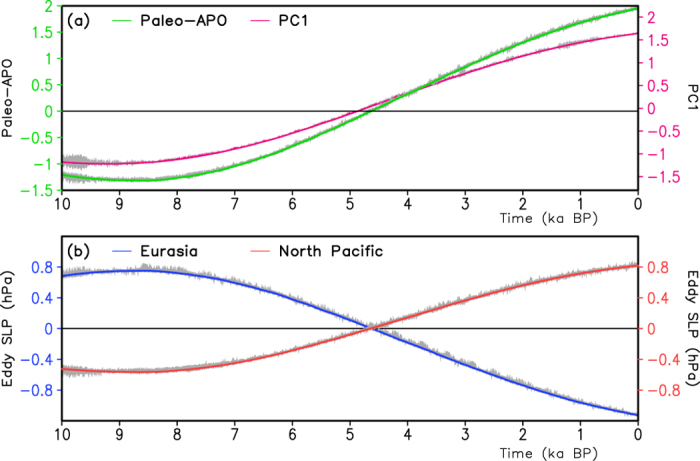
(**a**) The paleo-APO index and PC1 in Fig. 2b and (**b**) 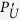 anomaly from climatological mean over the Eurasian continent and the North Pacific Ocean over the past 10 ka.

Previous studies have shown that differences in SLP between Asia and the North Pacific may be used to indicate the contrasts in surface air temperature between these two regions^15^,^16^,^17^,^18^. In fact, the anomalous patterns shown in both Fig. 2a,b are similar to the eddy SLP anomalies associated with APO in the present-day climate (during 1948–2014), in which the APO is a leading mode of the tropospheric eddy temperature and its index is defined as the reference^19^. In the present day (Fig. 4), negative/positive anomalies of SLP appear over North Africa and Asia/the North Pacific and North America, which indicate weaker than normal Asian landmass low pressure and stronger than normal North Pacific high pressure. Therefore, the anomalous pattern of SLP in the UVic Model (shown in Fig. 2a,b) is called the paleo-APO pattern. Referring to the positions of positive and negative SLP anomalies in Fig. 2a,b, we define the paleo-APO index as a difference in the simulated normalized 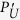 between the North Pacific (150°E–110°W, 15°–60°N) and the Eurasian landmass (10°–110°E, 15°–60°N). The summer paleo-APO index shows an increasing trend, similar to the variability of PC1 (Fig. 5a). This result suggests that the paleo-APO index may reasonably indicate a variation in the leading mode of the eddy SLP. Meanwhile, the summer solar radiation insolation along 30°N (Fig. 1b) changes from the top to the valley. Thus, when the NH summer incoming solar radiation decreases during the Holocene, the surface thermal contrast between Eurasia and the North Pacific increases.

Figure 5b shows the climatological anomalies of the 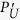 over Eurasia and the North Pacific during the summer. The Eurasian 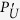 varies from −0.5 hPa to 0.75 hPa and the North Pacific 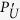 changes from 0.6 hPa to −1 hPa, which indicates a larger varying range over Eurasia (1.6 hPa) than over the North Pacific (1.25 hPa) and also suggests a larger response of the eddy SLP over Eurasia compared with that over the North Pacific. Meanwhile, the 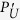 shows a decreasing trend over Eurasia and an increasing trend over the North Pacific during the Holocene, which also leads to an increased trend of the paleo-APO index during the Holocene. Compared to the North Pacific, therefore, the land response to the orbital forcing possibly plays a more important role in modulating the zonal thermal contrast between Eurasia and the North Pacific.

**Figure 6:**
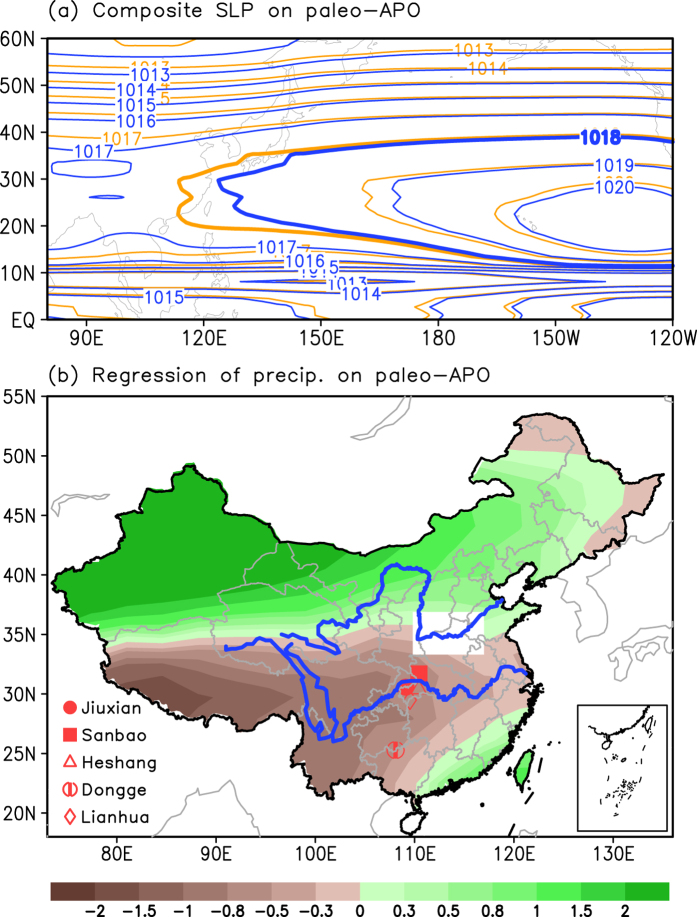
(**a**) Composite SLP filed in the high (during the period 0.09–0 ka BP; orange) and low (during the period 10–9.91 ka BP; blue) paleo-APO index years, in which the contours of 1018 hPa are thickened to denote the domain of the North Pacific high pressure zone. (**b**) Regression of summer precipitation (unit: mm) over eastern China on the paleo-APO index, in which the locations of stalagmite proxy data at the Jiuxian Cave, Sanbao Cave, Heshang Cave Dongge Cave, and Lianhua Cave are also given and values exceeding significance at the 95% confidence level are plotted. This figure is generated by the Grid Analysis and Display System (GrADS) Version 2.0.1.oga.1 Copyright (**c**) 1988–2011 by Brian Doty and the Institute for Global Environment and Society (IGES) (ftp://cola.gmu.edu/grads/2.0/old/).

Figure 6a depicts the composite summer model SLP in the higher and lower paleo-APO indices. The North Pacific high pressure has the central values of 1020 hPa in the higher and lower paleo-APO years, but exhibits a larger domain in the higher paleo-APO years than in the lower paleo-APO years, which suggests a stronger North Pacific high pressure in the higher paleo-APO phase. The western boundary of the North Pacific high pressure in the higher paleo-APO phase moves westward along 115°E compared with the lower paleo-APO phase. That is, the increasing (decreasing) trend of the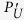 over the North Pacific (Eurasia) implies a stronger and more westward high pressure over the North Pacific.

The analyses presented here show that the response of the eddy SLP to orbital forcing displays an APO-like pattern, with positive and negative anomalies of the 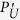 in the North Pacific and Eurasia, respectively. Corresponding to the NH’s summer reduced incoming solar radiation during the Holocene, the paleo-APO index shows an increasing trend. The response of the Eurasian landmass plays a more important role due to a (an) decreasing (increasing) trend of the 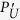 over Eurasia (the North Pacific). Meanwhile, the increasing trend of the 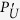 over the North Pacific indicates a stronger and more westward high pressure over the North Pacific.

### Summer hydrology anomalies over eastern China associated with the paleo-APO at the precession

Many studies on the present-day climate have shown that the thermal contrasts between the East Asian landmass and its adjacent oceans exerts a strong influence on the hydrological climate in the EASM region and that a strong and westward high pressure over the North Pacific could strengthen the northward transport of water vapor over the EASM region, with more rainfall over North China and less rainfall over the Yangtze River valleys^3^,^15^,^19^,^71^,^72^. When the paleo-APO index is high, the domain (central intensity) of the North Pacific high pressure is larger (stronger) compared with that in the low paleo-APO index, and the western part of the North Pacific high pressure moves westward (Fig. 6a). This variation in the North Pacific high pressure favors transport of water moisture toward northern China^73^. As a result, precipitation is often less over the Yangtze River. At the precession, the variability of thermal contrasts indicated by the paleo-APO index is also associated with precipitation over eastern China. Figure 6b shows the summer rainfall anomalies over eastern China that are associated with the paleo-APO index. In this figure, the rainfall anomalies present a meridional “positive-negative-positive” anomalous pattern, with negative anomalies along the Yangtze River and over Southwest China and positive rainfall anomalies over North China. This anomalous pattern is also a major feature of the leading EOF mode of summer rainfall over eastern China during the Holocene (not shown).

**Figure 7:**
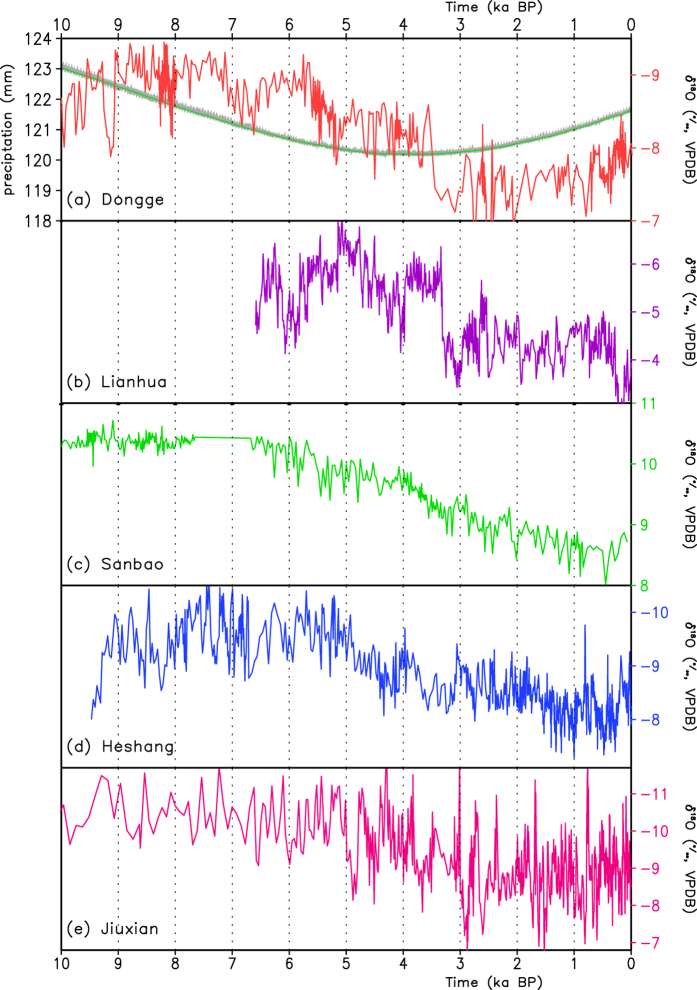
Stalagmite δ^18^O records and the simulated summer precipitation over the middle reach of the Yangtze River and southwestern China. (**a**) The Dongge Cave δ^18^O record (red) and the simulated July precipitation over 100°E–112°E, 22°N–32°N (green; mm), (**b**) the Lianhua Cave δ^18^O record, (**c**) the Sanbao Cave δ^18^O record, (**d**) the Heshang Cave δ^18^O record, and (**e**) the Jiuxian Cave δ^18^O record.

Recent studies have suggested that the speleothem δ^18^O records in eastern China represent the changes in the hydrological climate and are mainly a result of the δ^18^O changes in precipitation. Hence, we compare the simulated rainfall with five speleothem δ^18^O records over Southwest China and the middle valley of the Yangtze River (100°–112°E, 22°–32°N), one key region of the EASM precipitation and an important path of moisture transport from the Indian Ocean (Fig. 7). The speleothem δ^18^O record at Dongge Cave (Fig. 7a) has generally shown a decreasing trend before a slight increase trend since 3.5 ka BP, which indicates the declining precipitation and the obvious decadal- and century-scale fluctuations. Similarly, the speleothem δ^18^O records at Lianhua Cave (Fig. 7b), Sanbao Cave (Fig. 7c), Heshang Cave (Fig. 7d), and Jiuxian Cave (Fig. 7e) also exhibit a downward trend during the Holocene. The simulated summer precipitation similarly presents a decreasing trend before 4 ka and an increasing trend afterwards, indicating wetter conditions in the lower paleo-APO years and drier conditions in the higher paleo-APO years, similar to the variation in the speleothem δ^18^O records. This result further supports the reliability of the simulation.

### Atmospheric circulation anomalies associated with the paleo-APO evolution

**Figure 8:**
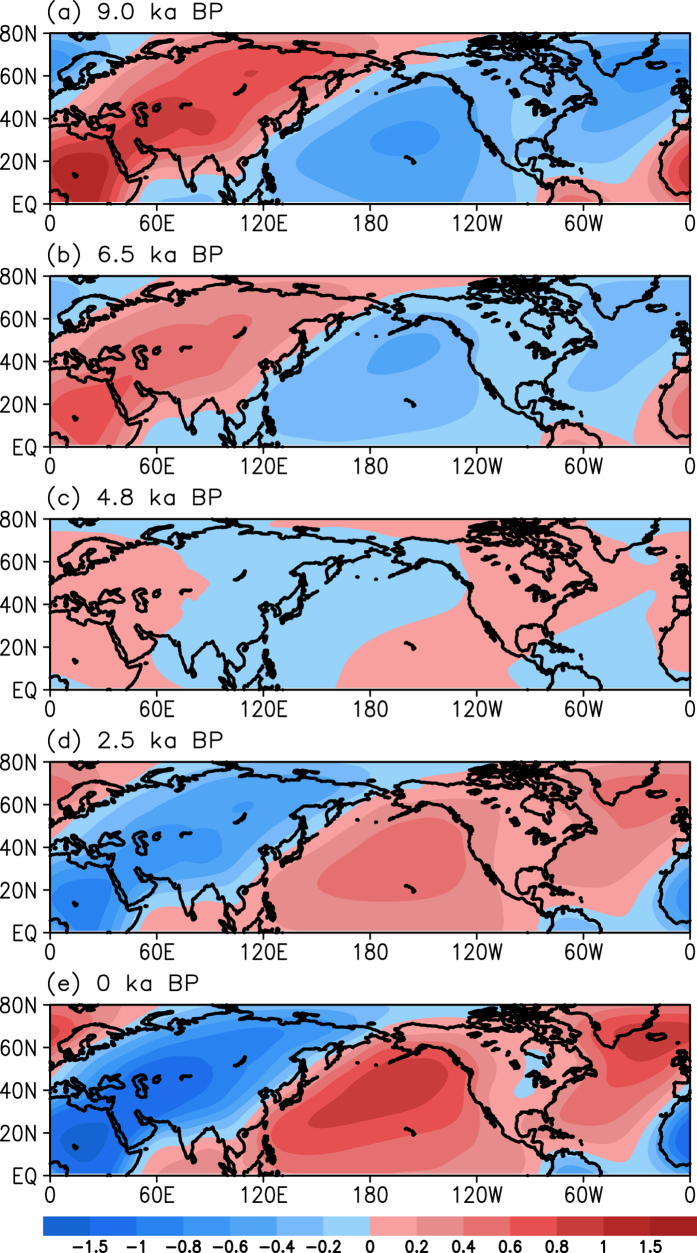
Anomalies in the ensemble mean summer eddy SLP (unit: hPa) from the climatological mean at 9 (**a**), 6.5 (**b**), 4.8 (**c**), 2.5 (**d**), and 0 (**e**) ka BP. This figure is generated by the Grid Analysis and Display System (GrADS) Version 2.0.1.oga.1 Copyright (**c**) 1988–2011 by Brian Doty and the Institute for Global Environment and Society (IGES) (ftp://cola.gmu.edu/grads/2.0/old/).

In this analysis, we use transient simulation to examine the evolution of the paleo-APO and associated atmospheric circulation and precipitation. It is seen in Fig. 5a that the paleo-APO index exhibits an increasing trend in approximately half of the precession period. According to the paleo-APO index, we chose 9.0, 6.5, 4.8, 2.5, and 0 ka BP to analyze the evolution of atmospheric circulation. Figure 8 shows the summer eddy SLP anomalies (from the climatological mean) at 9.0, 6.5, 4.8, 2.5 and 0 ka BP. At 9 ka BP (Fig. 8a), there are negative SLP anomalies over the North Pacific, with a maximum negative value of −0.8 hPa, and there are positive SLP anomalies over Eurasia, with a maximum value of 1.5 hPa. These features suggest a stronger negative phase of the paleo-APO. At 6.5 ka BP (Fig. 8b), the eddy SLP anomalies depict a similar pattern to that at 9 ka BP, with relatively lower SLP anomalies over both Eurasia and the North Pacific, indicating a moderate negative phase of the paleo-APO. At 4.8 ka BP (Fig. 8c), small positive eddy anomalies move from Eurasia to West Europe, North Africa, North America, and the tropical eastern Pacific, whereas the other regions are covered by small negative anomalies. This distribution of the eddy SLP anomalies suggests a very weak paleo-APO phase at 4.8 ka BP. The eddy SLP anomalies indicate a moderate positive phase of the paleo-APO at 2.5 ka BP (Fig. 8d), which is opposite to the value at 6.5 ka BP. At the present day (Fig. 8e), the paleo-APO further intensifies, with larger anomalies over the North Pacific-North Atlantic region and Eurasia. Thus, the paleo-APO changes from a negative phase to a positive one during the Holocene.

**Figure 9:**
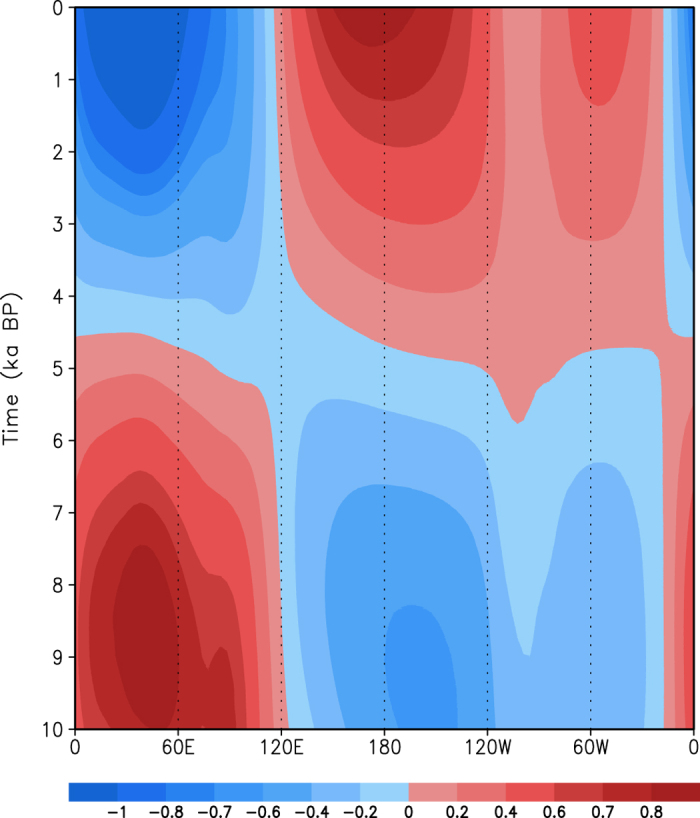
Longitude-time cross-section of the eddy SLP anomalies (unit: hPa) from the climatological mean along 30°N.

Figure 9 shows the evolution of the eddy SLP anomalies along 30°N during the Holocene. There is an anomalous SLP center at the longitudes 30°W–120°E during 10–5.5 ka, with a maximum positive value of 1 hPa along 50°E at 9 ka BP. As time goes on, the positive anomalies of the eddy SLP decrease and turn to the negative ones after 5.5 ka BP. The strongest negative anomalies appear along 40°E at the present day. Over the North African and Asian landmasses along 30°N, the anomalous SLP center moves slightly westward during the Holocene. During the period 10–5 ka, negative SLP anomalies appears over the longitudes 120°E–30°W, with a center of −0.6 hPa along 140°W–180° and a relatively weak center along 60°W. Similarly, the negative SLP anomalies decrease, turn to positive values, and reach the present-day largest values. The negative center at 150°W at 10 ka BP turns to a positive one at 170°E at the present day, which suggests a westward shift of SLP anomalies over the North Pacific. However, no obvious shift in SLP anomalies is seen over the North Atlantic during the Holocene.

**Figure 10:**
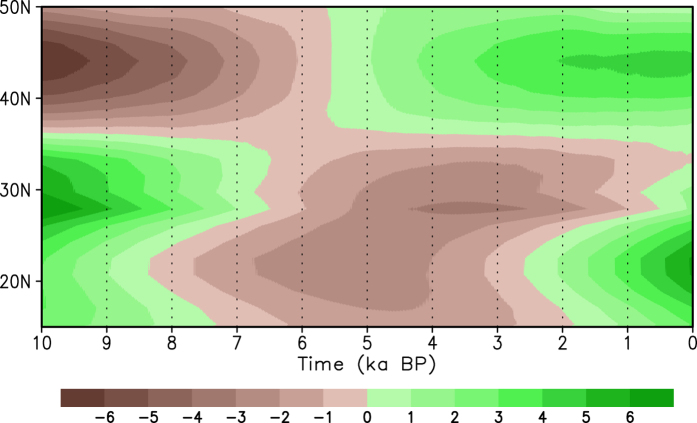
Latitude-time cross-section of summer precipitation anomalies (unit: mm) from the climatological mean along longitude 110°E.

We also display the time-latitude cross section of summer mean precipitation anomalies (from the climatological mean) over eastern China along 110°E (Fig. 10). At 10 ka BP, there is a meridional dipole pattern of rainfall anomalies, with positive anomalies south of 36°N and negative anomalies at higher latitudes. Afterwards, the positive and negative anomalies gradually weaken and turn to negative and positive values, respectively, in which negative rainfall anomalies from southern China show a northward propagation to 36°N. An opposite dipole appears between 5.5 ka BP and 2.5 ka BP. After that, a triple pattern appears over eastern China, with positive precipitation anomalies south of 30°N and north of 36°N and negative precipitation anomalies near the Yangtze River. These results indicate that when the paleo-APO pattern moves from a negative phase to a positive one during the Holocene, the rainfall belt exhibits a northward propagation from southern China.

## Summary and Discussion

We employed the intermediate-complexity UVic Model to investigate the response of summer large-scale circulation over Eurasia and the North Pacific and the associated precipitation anomalies over eastern China to the transient orbit forcing during the Holocene. A decrease in the NH summer incoming solar radiation is corresponded to an out-of-phase relationship of the summer eddy SLP over the Asian-Pacific region, with positive anomalies over the Asia-Europe-African continent and negative anomalies over the North Pacific and North America, which reflect the leading mode of the eddy SLP during the Holocene. This anomalous pattern is similar to that associated with the present-day APO, called the paleo-APO.

The paleo-APO index exhibits an upward trend during the Holocene, which indicates an increase in the zonal thermal contrast between the North Pacific and Eurasia. Such a trend is possibly due to a decrease in the 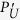 over the Eurasian landmass and an increase in the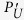 over the North Pacific, and there is a larger varying range over Eurasia than over the North Pacific. This result suggests that the response of atmospheric circulation over the Eurasian landmass to orbital forcing is larger than that over the North Pacific, possibly playing a more important role in modulating the zonal thermal contrast between Eurasia and the North Pacific.

A higher paleo-APO index during the Holocene is corresponded to a stronger high-pressure system over the North Pacific that moves more westward, which favors the northward transport of water vapor toward northern China. Accordingly, there is more precipitation over northern China and less rainfall over the middle and lower reaches of the Yangtze River during the summer. This decrease in the simulated precipitation over the middle and lower reaches of the Yangtze River is supported by the local speleothem proxy data during the Holocene.

The paleo-APO experiences a half-precession cycle during the Holocene. During the early Holocene, the paleo-APO pattern presents a negative phase with positive SLP anomalies over the Eurasian landmass and negative SLP anomalies over the North Pacific. These positive (negative) anomalies over the Eurasian landmass (North Pacific and North Atlantic) decrease and turn into negative (positive) anomalies, which indicate a change in the paleo-APO pattern from a negative phase to a positive one. The negative centers over the North Africa-Asian region and the North Pacific move westward. Meanwhile, the rainfall belt exhibits a northward propagation from southern China, and a meridional dipole pattern of rainfall anomalies during the early Holocene gradually turns into the present-day triple pattern.

Zhou^74^ evaluated the performance of 33 models of the Coupled Model Intercomparison Project Phase 5 (CMIP5) in simulating the summer APO, and projected its variation under warming climate scenarios (RCP4.5 and RCP8.5). His findings suggested that the intensity of APO under a warming climate is weaker than the present day. Similarly, with the reduced trend of incoming solar radiation in the Holocene summer, the paleo-APO index exhibits an increasing trend, which is also supported by Zhou’s results. The summer precipitation anomalies over eastern China associated with the APO were investigated using the reanalysis and gauge data^19^. The result exhibits more rainfall anomalies over northern China and less rainfall over the Yangtze River. This relationship between APO and summer precipitation over eastern China occurs not only during 1900–2009^20^ and past 150 years^71^ but also throughout the past millennium^21^,^22^,^23^,^24^,^75^. Summer rainfall anomalies associated with the paleo-APO during the Holocene also exhibit a similar pattern, which implies that this relationship between APO and summer rainfall over eastern China also occurs on a longer precessional scale. Furthermore, variations in the paleo-APO and associated rainfall over eastern China also need more analyses using the General Circulation Model to better understand the underlying mechanisms and processes.

Ice-sheet changes are often considered as an important external forcing factor of climate change. They experienced a rapid change in the early Holocene (Fig. S2a). At that time, the ice-sheet covered the northeast portion of North America (Fig. S2b) and exerted a strong influence on climate change by changing the surface albedo and the solar insolation^76^. Some studies have shown that temperature anomalies caused by ice-sheet forcing represent planetary-scale waves in the extratropical northern hemispheric in winter and annual mean fields^77^. However, summer temperature anomalies forced by ice sheets are confined in the northeastern part of North America and the northern part of the North Atlantic, which implies a small effect of the ice-sheet forcing on summer temperature over the extratropical Asian-Pacific sector^77^. In this study, we further examine the impacts of ice-sheets in the early Holocene on the paleo-APO pattern and its variability by performing a simulation under both orbital and ice-sheet forcings. The results indicate that the leading mode of the summer eddy SLP under both orbital and ice-sheet forcings (Fig. S3a) are very similar to that under the orbital forcing alone except that the positive values over the northern part of the North Atlantic become larger compared to the ice-sheet forcing (Fig. 2b). The PC of the leading mode (Fig. S3b) generally exhibits an increasing trend that is consistent with that in Fig. 2c, although with a weak fluctuation in the early Holocene. Our simulation further supports that ice sheet changes in the early Holocene have a weak influence on the summer extratropical paleo-APO pattern.

## Additional Information

**How to cite this article**: Xiao, D. *et al*. Responses of the summer Asian-Pacific zonal thermal contrast and the associated evolution of atmospheric circulation to transient orbital changes during the Holocene. *Sci. Rep.* **6**, 35816; doi: 10.1038/srep35816 (2016).

## Supplementary Material

Supplementary Information
