# Supplementary material for: Responses of the summer Asian-Pacific zonal thermal contrast and the associated evolution of atmospheric circulation to transient orbital changes during the Holocene
Source: Sci Rep. 2016 Oct 25;6:35816. doi: 10.1038/srep35816 (PMC5078810; doi:10.1038/srep35816)
Supplement: Supplementary Information [file srep35816-s1.pdf]

Responses of the summer Asian-Pacific zonal thermal contrast and the associated evolution of atmospheric circulation to transient orbital changes during the Holocene

Dong Xiao[1,2], Ping Zhao[1,3], Yue Wang[4], and Xiuji Zhou[1]

[1]State Key Laboratory of Severe Weather, Chinese Academy of Meteorological Sciences, Beijing 100081, China
[2]Institute of Climate Systems, Chinese Academy of Meteorological Sciences, Beijing 100081, China
[3]Collaborative Innovation Center on Forecast and Evaluation of Meteorological Disasters, Nanjing University of Information Science and Technology, Nanjing, 210044, China
[4]State Key Laboratory of Marine Geology, Tongji University, Shanghai, 200092, China

Corresponding author: Ping Zhao

E-mail: zhaoping@camscma.cn

Figure captions

Figure S1. (a) The reconstructed July temperature over middle and western parts of U.S. (green) and the simulated July SLAT (red) over 100°W–120°W, 30°N–40°N. (b) The reconstructed July temperature (green) and the simulated July SLAT (red) over West Europe (15°E–20°E, 65°N–70°N). The gray shading denotes the spread from ensemble mean.

Figure S2. (a) Temporal curves of the ice-sheet accumulative elevation difference (unit: 1000 m) from the present day, and (b) the distribution of the ice-sheet elevation difference (unit: m) from the present day at the 10 ka BP. These figures are generated by Grid Analysis and Display System (GrADS) Version 2.0.1.oga.1 Copyright (c) 1988-2011 by Brian Doty and the Institute for Global Environment and Society (IGES) (ftp://cola.gmu.edu/grads/2.0/old/).

Figure S3. (a) The leading EOF mode of summer eddy SLP under both orbital and ice-sheet forcings and (b) its time series (green) and the time series under the orbital forcing only (black). These figures are generated by Grid Analysis and Display System (GrADS) Version 2.0.1.oga.1 Copyright (c) 1988-2011 by Brian Doty and the Institute for Global Environment and Society (IGES) (ftp://cola.gmu.edu/grads/2.0/old/).

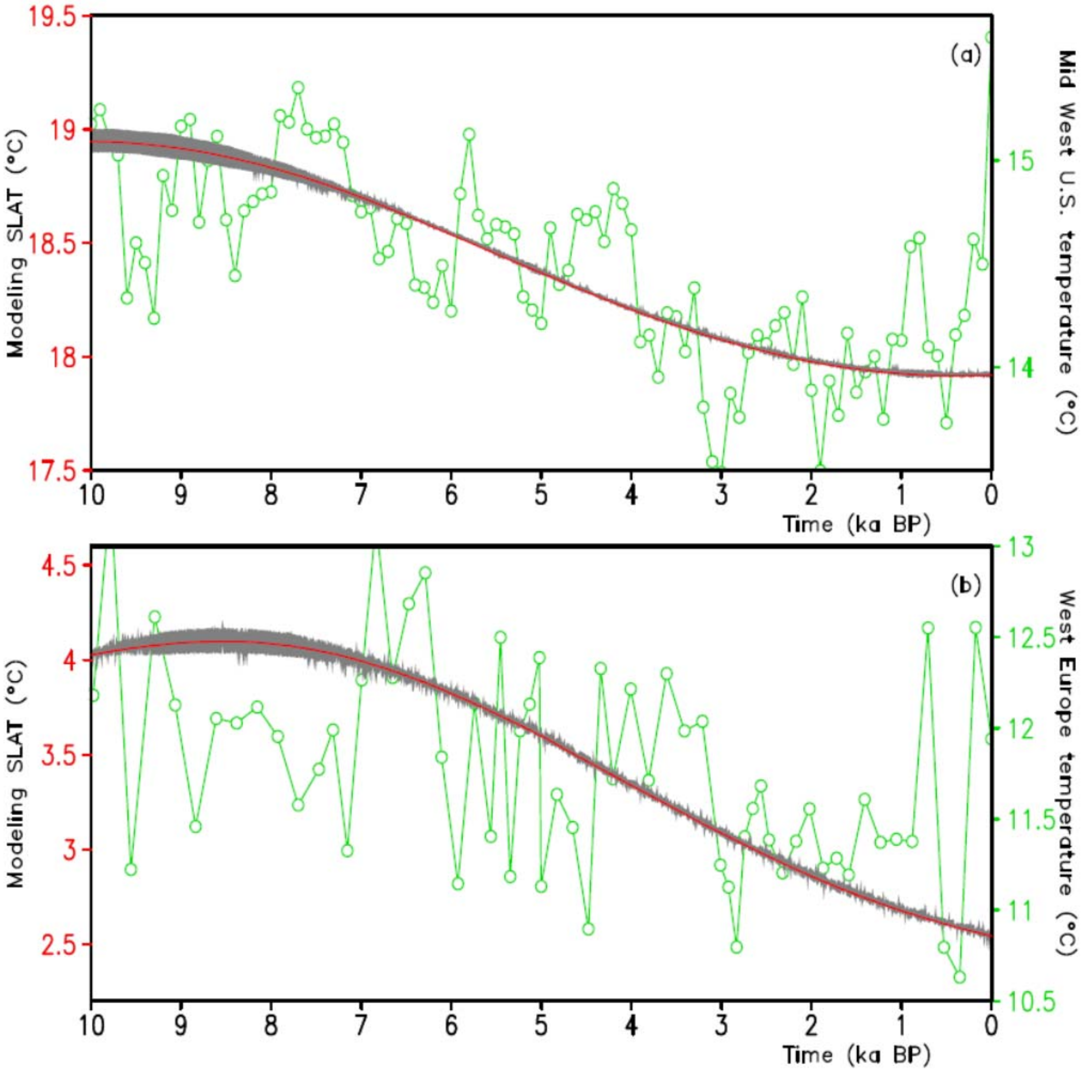

Figure S1. (a) The reconstructed July temperature over middle and western parts of U.S. (green) and the simulated July SLAT (red) over 100°W–120°W, 30°N–40°N. (b) The reconstructed July temperature (green) and the simulated July SLAT (red) over West Europe (15°E–20°E, 65°N–70°N). The gray shading denotes the spread from ensemble mean.

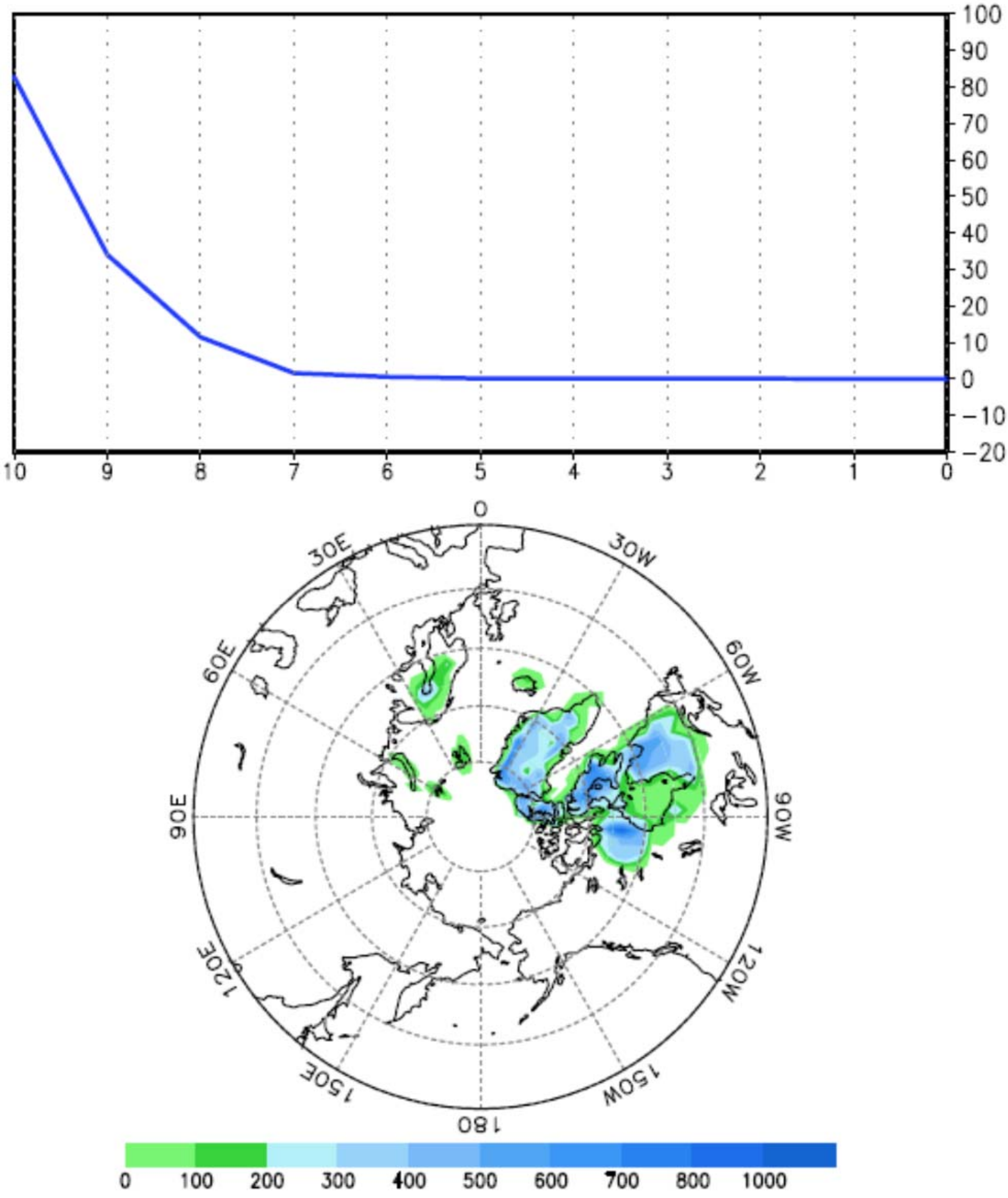

Figure S2. (a) Temporal curves of the ice-sheet accumulative elevation difference (unit: 1000 m) from the present day, and (b) the distribution of the ice-sheet elevation difference (unit: m) from the present day at the 10 ka BP. These figures are generated by Grid Analysis and Display System (GrADS) Version 2.0.1.oga.1 Copyright (c) 1988-2011 by Brian Doty and the Institute for Global Environment and Society (IGES) (ftp://cola.gmu.edu/grads/2.0/old/).

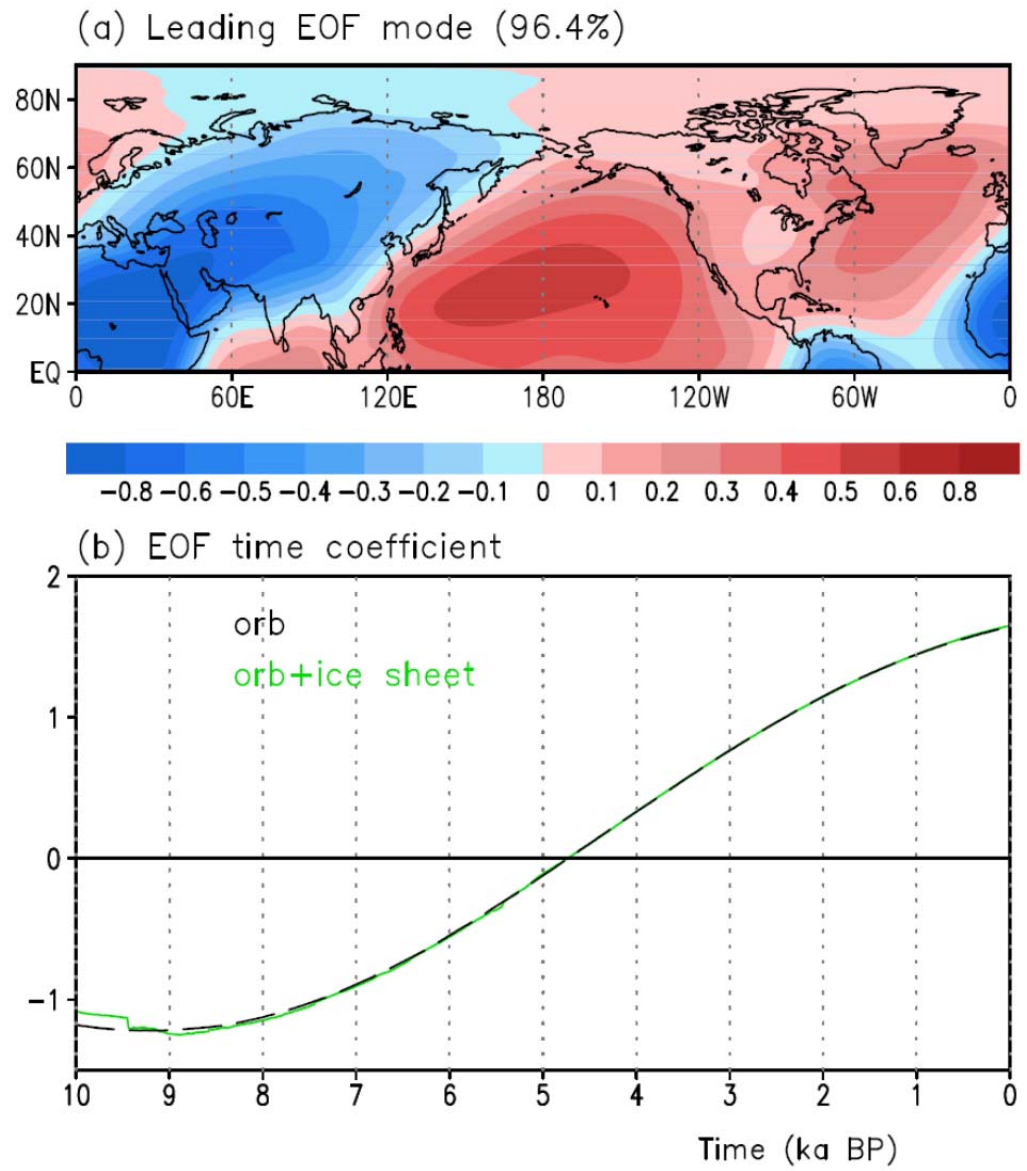

Figure S3. (a) The leading EOF mode of summer eddy SLP under both orbital and ice-sheet forcings and (b) its time series (green) and the time series under the orbital forcing only (black). These figures are generated by Grid Analysis and Display System (GrADS) Version 2.0.1.oga.1 Copyright (c) 1988-2011 by Brian Doty and the Institute for Global Environment and Society (IGES) (ftp://cola.gmu.edu/grads/2.0/old/).
